# Supplementary material for: Alternative reproductive strategies in black-winged territorial males of Paraphlebia zoe (Odonata, Thaumatoneuridae)
Source: PeerJ. 2019 Feb 20;7:e6489. doi: 10.7717/peerj.6489 (PMC6387578; doi:10.7717/peerj.6489)
Supplement: Table S1 — The notation for models follows Lebreton et al. (1992) , where g is group (three types of males and females) and t is time. The effect of body length (length) was included as an individual covariate in some models, sometimes as a quadratic effect (length2). Estimates corrected for c-hat = 1.007. [file peerj-07-6489-s001.docx]

Table S1. Model selection using QAICc. The notation for models follows Lebreton et al (1992), where *g* is group (three types of males and females) and *t* is time. The effect of body length (*length*) was included as an individual covariate in some models, sometimes as a quadratic effect (*length2*). Estimates corrected for c-hat= 1.007.

| Model | | AICc | Delta AICc | AICc Weights | Model Likeli-hood | Num. Par | Deviance |
| --- | --- | --- | --- | --- | --- | --- | --- |
| {Phi(g*length) p(g*t)}-common intercept} | 2275.292 | | 0.000 | 0.554 | 1 | 78 | 2103.751 |
| {Phi(g*length, length2) p(g*t)} | 2276.679 | | 1.386 | 0.277 | 0.500 | 81 | 2097.863 |
| {Phi(g*length) p(g*t)}-different intercept} | 2277.657 | | 2.364 | 0.170 | 0.307 | 81 | 2098.841 |
| {Phi(g) p(g*t)} | 2334.548 | | 59.256 | 0 | 0 | 100 | 2108.348 |
| {Phi(g* length) p(g)} | 2352.865 | | 77.573 | 0 | 0 | 9 | 2334.656 |
| {Phi(.) p(g*t)} | 2354.911 | | 79.619 | 0 | 0 | 97 | 2136.348 |
| {Phi(g) p(g)} | 2356.177 | | 80.885 | 0 | 0 | 8 | 2340.010 |
| {Phi(t) p(g*t)} | 2373.586 | | 98.294 | 0 | 0 | 119 | 2097.608 |
| {Phi(.) p(g)} | 2376.416 | | 101.124 | 0 | 0 | 5 | 2366.347 |
| {Phi(t) p(g)} | 2378.799 | | 103.507 | 0 | 0 | 28 | 2320.872 |
| {Phi(g) p(t)} | 2447.020 | | 171.727 | 0 | 0 | 28 | 2389.093 |
| {Phi(g*t) p(g)} | 2477.373 | | 202.080 | 0 | 0 | 100 | 2251.173 |
| {Phi(g*t) p(g*t)} | 2506.473 | | 231.180 | 0 | 0 | 184 | 2039.375 |
| {Phi(t) p(t)} | 2519.769 | | 244.477 | 0 | 0 | 47 | 2420.293 |
| {Phi(g*length) p(.)}-common intercept} | 2521.064 | | 245.772 | 0 | 0 | 6 | 2508.967 |
| {Phi(.) p(t)} | 2524.971 | | 249.678 | 0 | 0 | 25 | 2473.434 |
| {Phi(g) p(.)} | 2525.945 | | 250.653 | 0 | 0 | 5 | 2515.876 |
| {Phi(g*t) p(t)} | 2557.411 | | 282.119 | 0 | 0 | 118 | 2284.115 |
| {Phi(t) p(.)} | 2598.931 | | 323.639 | 0 | 0 | 25 | 2547.395 |
| {Phi(.) p(.)} | 2611.643 | | 336.350 | 0 | 0 | 2 | 2607.629 |
| {Phi(g*t) p(.)} | 2630.678 | | 355.386 | 0 | 0 | 97 | 2412.115 |
