# Supplementary material for: Alternative reproductive strategies in black-winged territorial males of Paraphlebia zoe (Odonata, Thaumatoneuridae)
Source: PeerJ. 2019 Feb 20;7:e6489. doi: 10.7717/peerj.6489 (PMC6387578; doi:10.7717/peerj.6489)
Supplement: Table S2 [file peerj-07-6489-s002.docx]

Table S2. Results of the GLM analysing the effects of phenotypic and environmental variables on copulation duration of *P. zoe*.

Regression analysis

Response variate: Copulation duration (Box-Cox transformed)

Fitted terms: Constant + Time of start + Temperature + asymmetry + Male body length + Number of individuals in the territory + Age in days

Summary of analysis

Source d.f. s.s. m.s. v.r. F pr.

Regression 6 0.1327 0.02211 0.99 0.446

Residual 36 0.8037 0.02232

Total 42 0.9363 0.02229

Estimates of parameters

Parameter estimate s.e. t(36) t pr.

Constant 1.346 0.343 3.93 <.001

Start 0.979 0.795 1.23 0.226

Temperature -0.0420 0.0201 -2.09 0.044

Asymmetry -0.048 0.179 -0.27 0.792

Male body length 0.00337 0.00358 0.94 0.352

Number ind. territory 0.0113 0.0147 0.77 0.446

Age -0.00036 0.00391 -0.09 0.928
