# Supplementary material for: Alternative reproductive strategies in black-winged territorial males of Paraphlebia zoe (Odonata, Thaumatoneuridae)
Source: PeerJ. 2019 Feb 20;7:e6489. doi: 10.7717/peerj.6489 (PMC6387578; doi:10.7717/peerj.6489)
Supplement: Table S3 [file peerj-07-6489-s003.docx]

Table S3. Results of the GLM analysing the effects of phenotypic and environmental variables on lifetime mating success of *P. zoe*.

Regression analysis

Response variate: Reproductive success

Distribution: Negative binomial with parameter k = 0.9259

Link function: Log-ratio with parameter k = 1.0000

Fitted terms: Constant + longevity + body length + number individuals territory + size fore black + size fore white + size hind black + size hind white + asymmetry + status

Summary of analysis

mean deviance approx

Source d.f. deviance deviance ratio F pr.

Regression 9 43.80 4.8663 10.02 <.001

Residual 158 76.76 0.4858

Total 167 120.56 0.7219

Dispersion parameter is estimated to be 0.486 from the residual deviance.

Estimates of parameters

Parameter estimate s.e. t(158) t pr.

Constant -3.29 3.04 -1.08 0.282

longevity 0.0415 0.0107 3.90 <.001

body length 0.239 0.683 0.35 0.727

number ind. territory 0.2320 0.0570 4.07 <.001

size fore black 1.71 3.74 0.46 0.648

size fore white -5.92 5.79 -1.02 0.308

size hind black -0.64 3.58 -0.18 0.859

size hind white 3.51 6.24 0.56 0.574

asymmetry -0.247 0.513 -0.48 0.630

status S -1.040 0.225 -4.62 <.001
