# Supplementary figures and images for: Alternative reproductive strategies in black-winged territorial males of Paraphlebia zoe (Odonata, Thaumatoneuridae)
Source: PeerJ. 2019 Feb 20;7:e6489. doi: 10.7717/peerj.6489 (PMC6387578; doi:10.7717/peerj.6489)

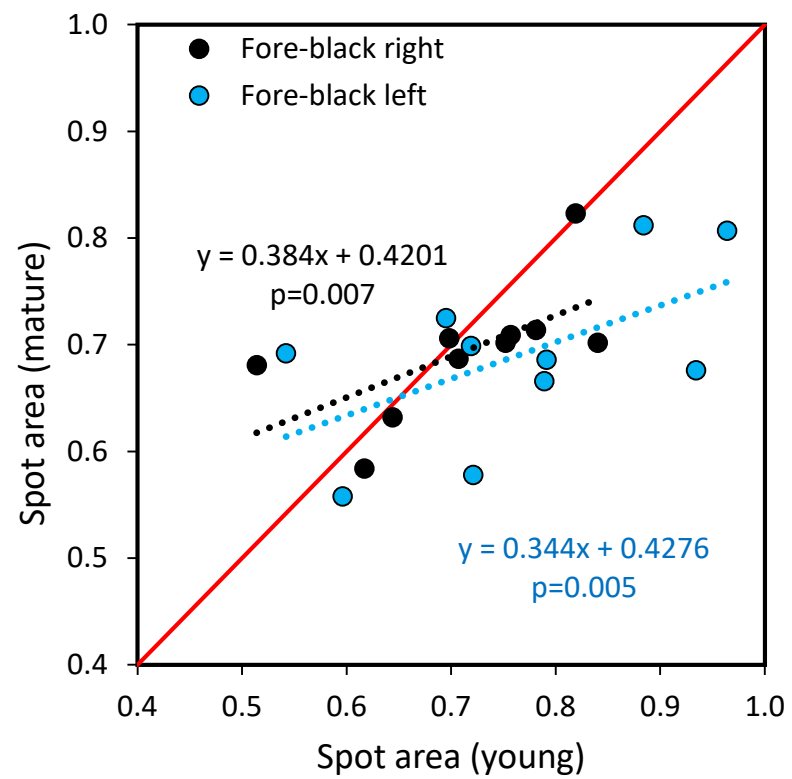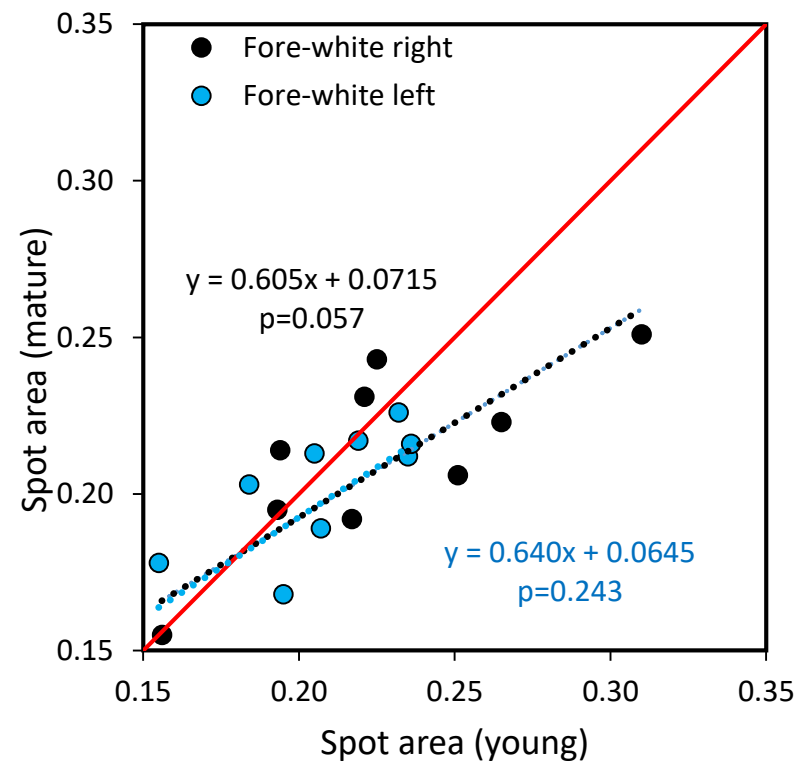

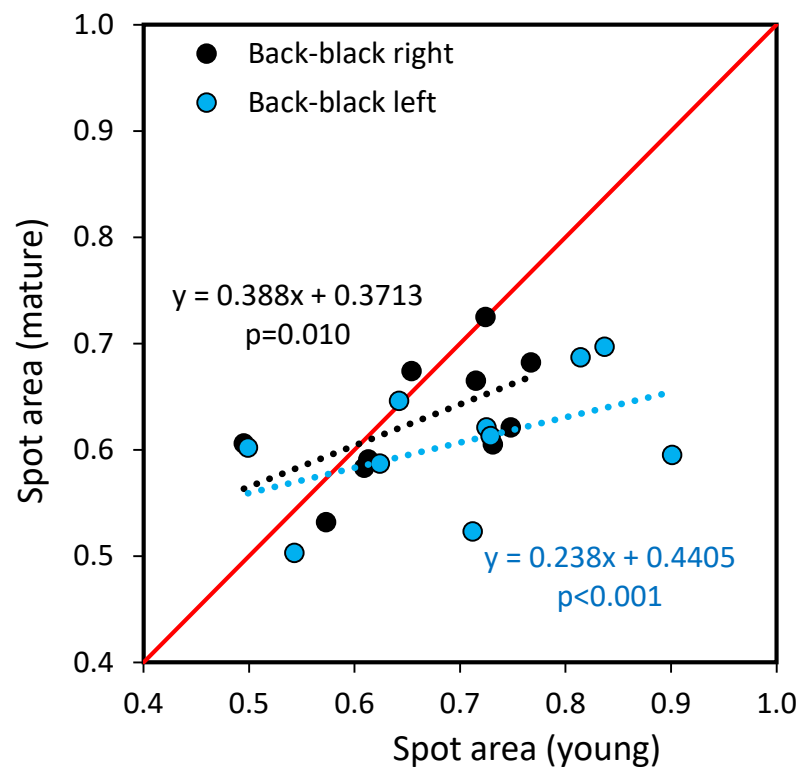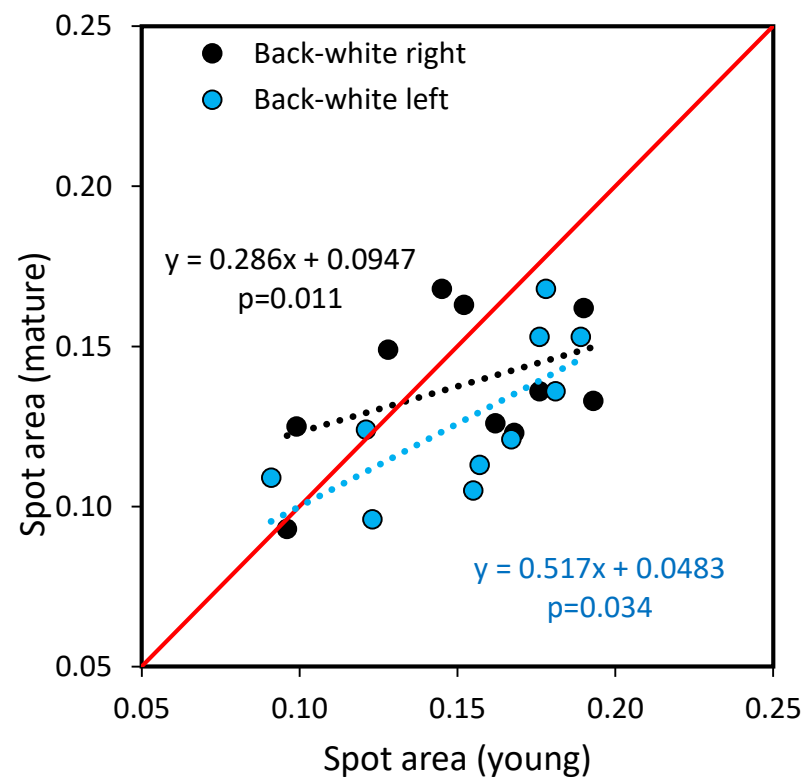

Supplement: Figure S1 — The original size is presented on the horizontal axis, and the final size in the vertical axis. If the spot does not change over ontogeny, the expectation is a line with a slope of 1 (red diagonal). Results indicate that all spots diminish in size, but the tendency is not significant for the white spot of the forewings. The equations for regressions of both sides are presented. The p-value tests the hypothesis of slope = 1. [file peerj-07-6489-s004.pdf]
